# Supplementary material for: Broad-spectrum applications of plant growth-promoting rhizobacteria (PGPR) across diverse crops and intricate planting systems
Source: Microbiol Spectr. 2025 Jan 22;13(3):e01879-24. doi: 10.1128/spectrum.01879-24 (PMC11878030; doi:10.1128/spectrum.01879-24)
Supplement: Supplemental material — Fig. S1; Table S1. [file spectrum.01879-24-s0001.docx]

**Table S1.** The formulation of the culture medium along with their corresponding sterilization parameters.

| Culture media types | Composition | Sterilization conditions |
| --- | --- | --- |
| Luria-bertani medium | Peptone 10.00 g, Yeast extract 5.00 g, NaCl 10.00 g, Agar 20.00 g, distilled water 1000 mL, pH 7.0-7.2 | 121 °C for 20 min |
| Luria-bertani liquid medium | Without agar, same composition as LB medium | Same as LB medium |
| Monkina medium | Glucose 10.00 g, (NH_4_)_2_SO_4_ 0.50 g, MgSO_4_·7H_2_O 0.30 g, NaCl 0.30 g, KCl 0.30 g, FeSO_4_ 0.03 g, MnSO_4_·H_2_O 0.03 g, distilled water 1000 mL | 115 °C for 30 min |
| Organic phosphorus liquid medium | 1000 mL Monkina medium with 0.40 g yeast extract, plus 0.20 g soluble lecithin; | Same as Monkina medium |
| Pikovskaya medium | Ca_3_(PO_4_)_2_ 5.00 g, Glucose 10.00 g, (NH_4_)_2_SO_4_ 0.50 g, NaCl 0.30 g, MgSO_4_·7H_2_O 0.30 g, KCl 0.30 g, MnSO_4_·H_2_O 0.03 g, FeSO_4_·H_2_O 0.03 g, distilled water 1000 mL, pH7.0-7.2 | 121 °C for 20 min |
| Liquid medium for potassium-solubilizing bacteria | Sucrose 10.00 g, Yeast extract 0.50 g, (NH_4_) _2_SO_4_ 1.00 g, Na_2_HPO_4_ 2.00 g, MgSO_4_·7H_2_O 0.50 g, CaCO_3_ 1.00 g, Potassium feldspar powder 1.00 g, distilled water 1000 mL | 121 °C for 20 min |
| Inorganic salt medium | (NH_4_)_2_SO_4_ 2.00 g; NaH_2_PO_4_ 0.50 g; K_2_HPO_4_ 0.50 g; MgSO_4_·7H_2_O 0.20 g; CaCl_2_·2H_2_O 0.10 g, distilled water 1000 mL | 121 °C for 20 min |


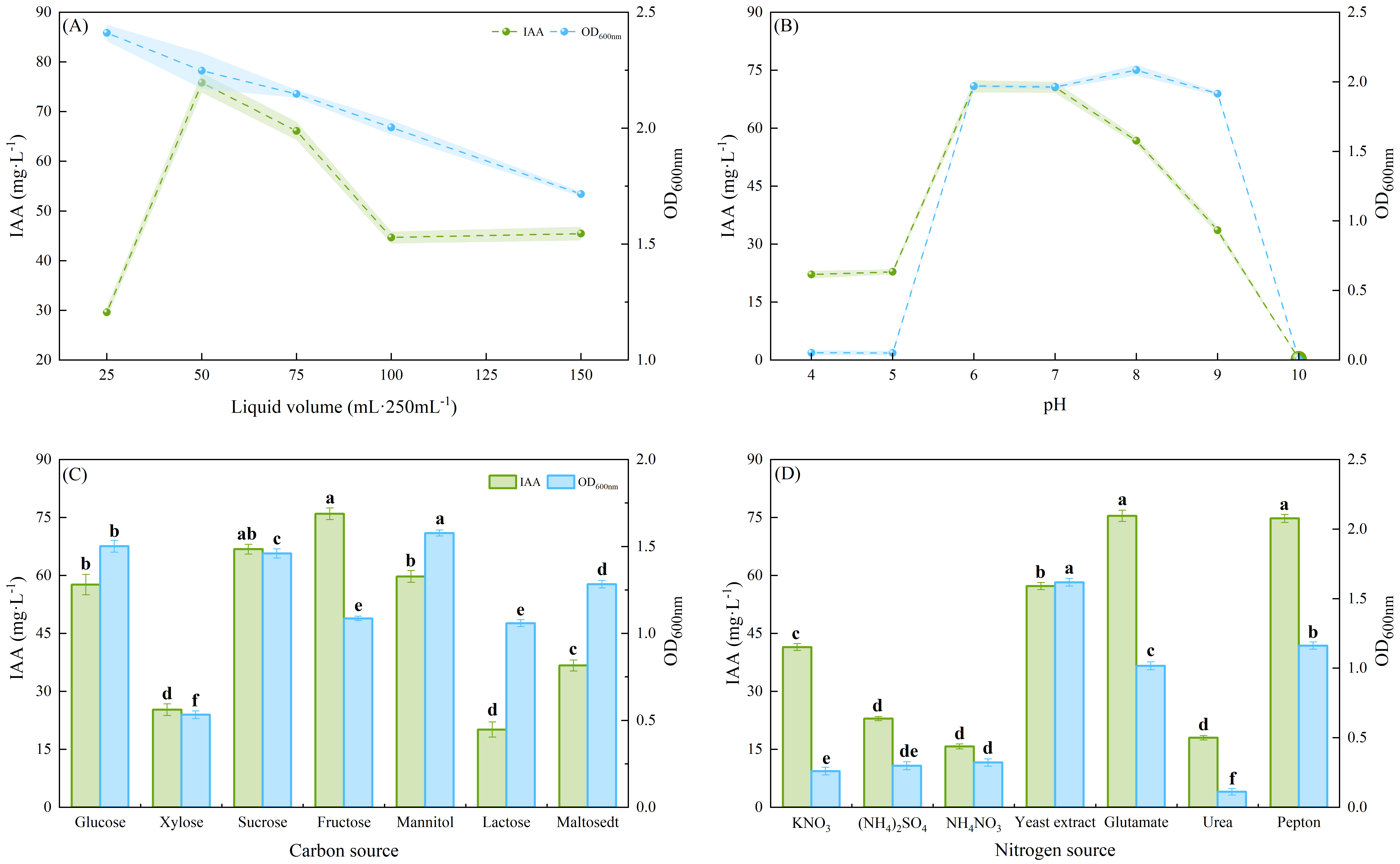


**Fig. S1** The effects of Liquid volume (A), pH (B), Carbon source (C), and Nitrogen source (D) on the production of IAA capability and growth status (OD_600nm_) of strain L8. Lowercase letters indicate significance (*P* ＜ 0.05) between different treatments.
